# The impact of alterations in lamin A on genome integrity

**DOI:** 10.1016/j.mrrev.2025.108577

**Published:** 2025-12-05

**Authors:** Alannah J. DiCintio, Alan S. Waldman

**Affiliations:** Department of Biological Sciences, University of South Carolina, Coker Life Sciences Building, 700 Sumter Street, Columbia, SC 29208, USA

**Keywords:** Nuclear lamina, Laminopathy, DNA Repair, Genome stability, Progeria, Aging, Progerin, ZMPSTE24

## Abstract

The maintenance of the genome in eukaryotic cells is dependent on the proper maintenance of the structure and function of the nuclear envelope which encases the genome. The nuclear envelope in higher eukaryotic cells is composed of the outer nuclear membrane, the inner nuclear membrane, and the nuclear lamina which resides just inside of the inner nuclear membrane. The nuclear lamina provides mechanical support to the nuclear envelope, plays essential roles in transport of molecules between the cytoplasm and the nucleus, and is pivotal in regulating global chromatin structure and three-dimensional nuclear architecture. Proper functioning of the nuclear lamina plays roles in regulating the cell cycle, transcription, RNA splicing, chromatin organization, DNA replication, and DNA repair. The nuclear lamina is conserved in metazoans and is composed of a meshwork of interwoven proteins called lamins, as well as lamin associated proteins. The protein known as lamin A is a vital constituent of the nuclear lamina. Alterations in lamin A, particularly those associated with disruptions in posttranslational processing of lamin A by zinc metallopeptidase ste24, have been linked to a variety of genetic disorders that give rise to genome instability and accelerated aging. This review will concentrate primarily on what has been learned about the dependency of effective DNA repair and DNA replication on a functional nuclear lamina, with particular emphasis on how modifications in the protein lamin A may corrupt a cell’s ability to maintain genome stability.

## 1. The nuclear lamina

The nucleus of eukaryotic cells houses the genome, and preservation of genome structure and function requires proper maintenance of the structure and function of the nuclear envelope (NE). The NE encases the nucleus, separating the nucleus from the cytoplasm, and in higher eukaryotes is composed of the outer nuclear membrane (ONM), the inner nuclear membrane (INM), and the nuclear lamina (NL) [1]. The NL is a filamentous meshwork that underlies the INM and plays important roles in nuclear architecture and function. The NL is conserved in metazoans and is a highly dynamic structure that is composed of interwoven proteins called lamins and lamin associated proteins [1–5]. A list of some of the lamin associated proteins and their functions can be found in Table 1. The lamin proteins, which constitute the principal components of the nuclear lamin, are evolutionary progenitors of intermediate filament (IF) proteins and are classified as type V IF proteins [22]. Lamins provide mechanical strength and stability to the nucleus and work with the lamin associated proteins to play roles in numerous nuclear functions. The NL determines the shape and size of the nucleus of a cell, which is vital to the load-bearing properties of the nucleus to provide it strength against stress [22]. Nuclear pore complexes, used for transporting macromolecules between the nucleus and cytoplasm, are embedded within the NE and the distribution of these complexes is anchored by lamins [22,24]. The NL also plays diverse roles in regulating the cell cycle, transcription, RNA splicing, global chromatin organization, DNA replication, and DNA repair [1–5,22,24–32]. We will focus on how alterations in the protein lamin A, a major component of the NL that is altered in several genetic disorders, may corrupt DNA replication and DNA repair.

## 2. Synthesis and processing of lamins

**Fig. 1.**
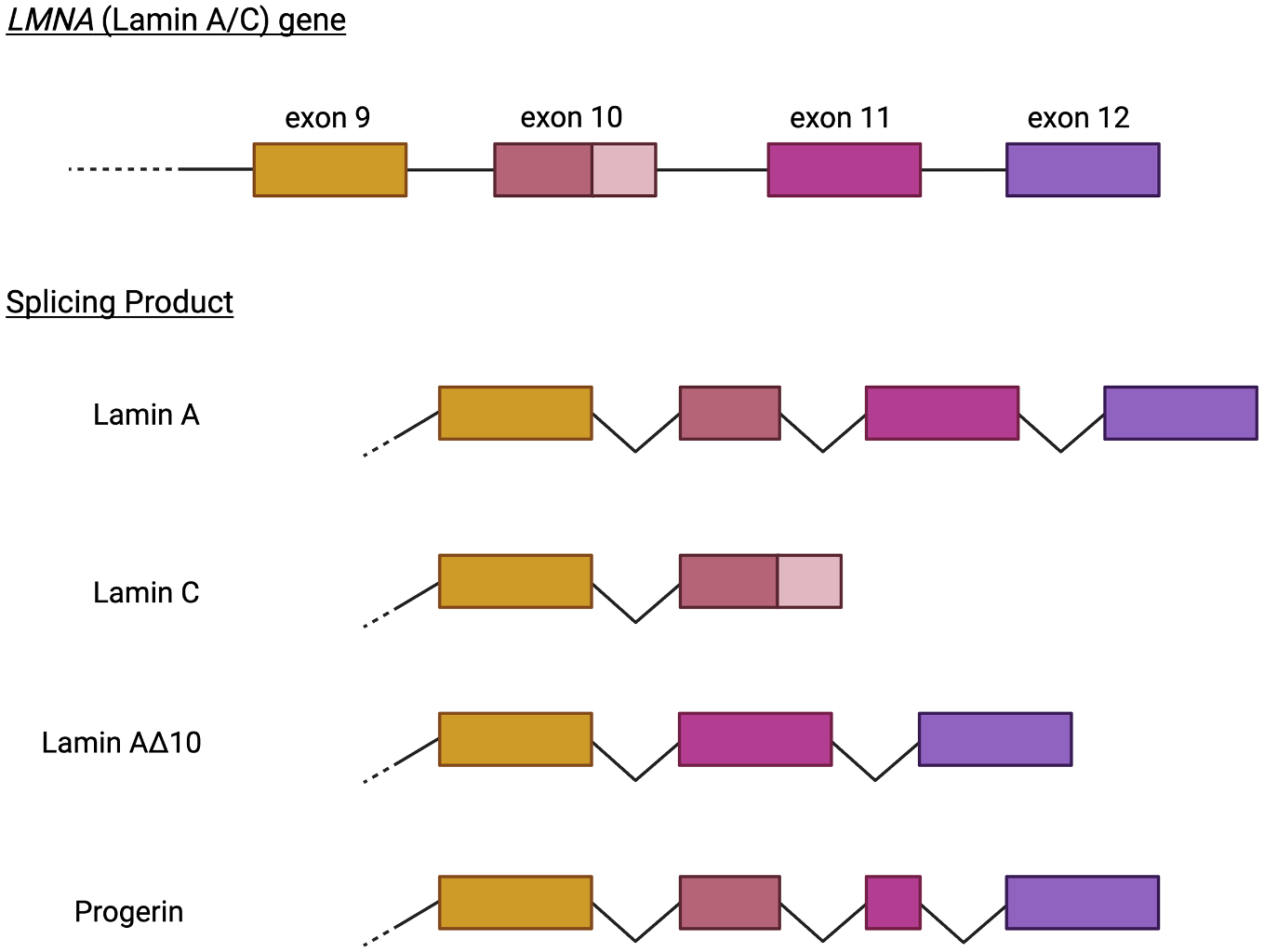
Splice variants of the LMNA gene. Differential splicing of the LMNA gene transcript normally leads to the production of protein products lamin A, lamin C, and lamin AΔ10. Activation of a cryptic splice site in exon 11 can lead to the production of a mutant protein known as progerin. See text for details. (This figure was created in Biorender and was adapted from Machiels et al. [39] and Singh et al. [34].).

As stated above, lamin proteins constitute major components of the NL. The two primary types of lamins are designated A-type and B-type [33]. Lamin B1 and lamin B2 are expressed in all animal cells, while lamin B3 is only expressed in spermatocytes [33,35,36]. A-type lamins are differentially expressed in somatic cells and their appearance in certain cell types is correlated with differentiation, with differentiated cells having higher expression of A-type lamins compared to undifferentiated stem cells [27,28,35,37]. The A family of lamins, which includes lamin A, lamin AΔ10, and lamin C, are all encoded by the *LMNA* gene via differential splicing of transcripts, as shown in Fig. 1 [38, 39]. Due to this alternative splicing, lamin C mRNA contains only exons 1–10, while lamin A mRNA additionally contains exons 11 and 12 (Fig. 1) [39]. Furthermore, the lamin C transcript contains additional sequence appended to exon 10 that is absent from the lamin A transcript. A variant of lamin A, known as lamin AΔ10, is missing exon 10 (Fig. 1) [38,39]. Lamins possess a nuclear localization signal in exon 7 and they are the only type of intermediate filament proteins that possess a CaaX motif [40–42]. The CaaX motif, which is located in exon 12, consists of a cysteine (C), two aliphatic amino acids (aa), and a terminal amino acid (X) and serves as the site of posttranslational modification for protein maturation to help lamin A associate with the nuclear membrane during the process of ultimately integrating into the NL [40–42]. Lamin C does not require posttranslational processing to become a mature protein, as it does not contain exon 12 and thus does not contain the CaaX motif [43]. However, lamin A and lamin AΔ10 do undergo necessary processing involving the CaaX motif to become mature proteins.

**Fig. 2.**
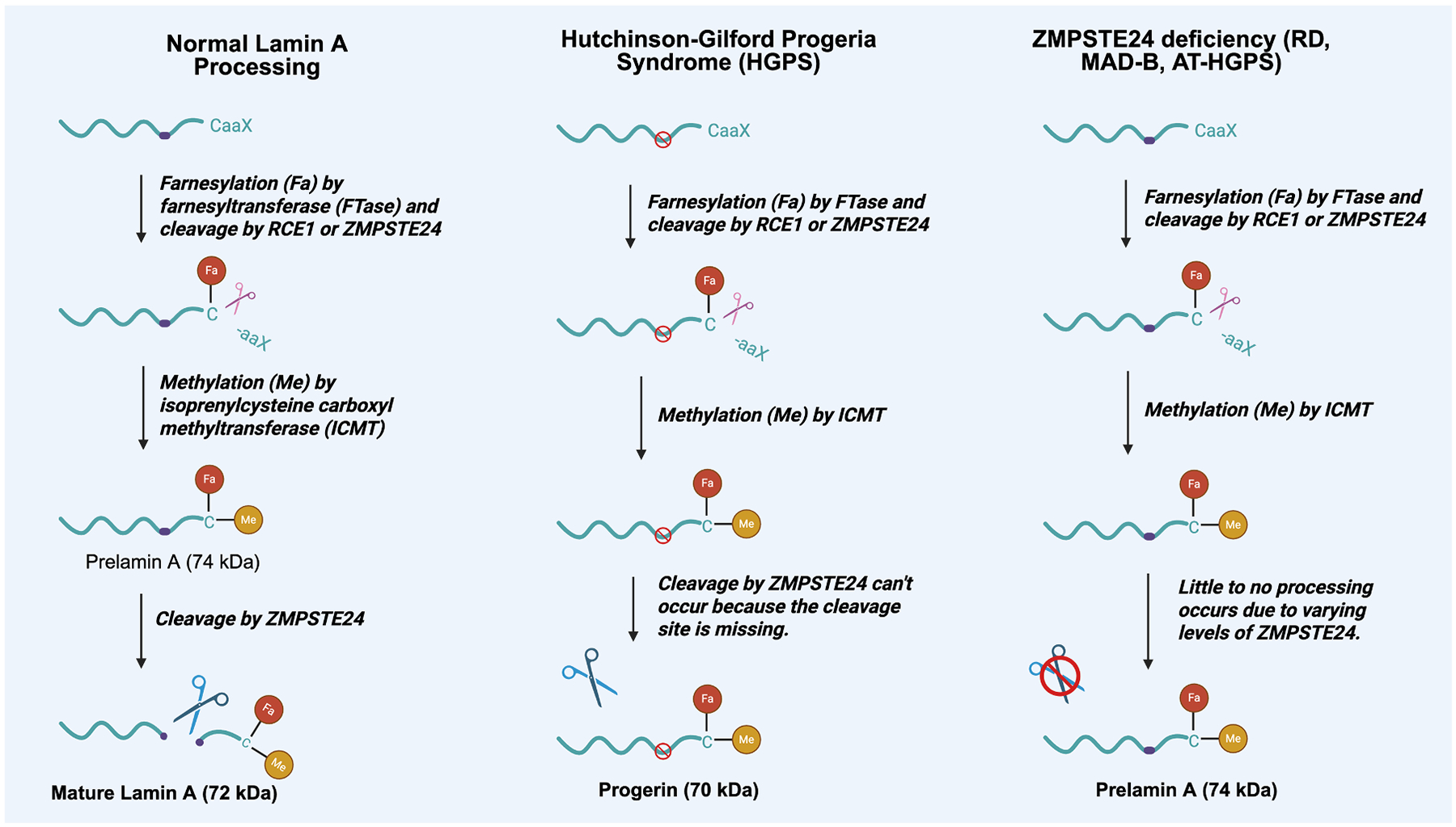
Processing pathways for normal and mutant forms of lamin A. The normal form of lamin A does not contain a farnesyl or methyl group following complete processing. Progerin, the mutant form of lamin A produced in HGPS, has a 50 amino acid deletion due to abnormal splicing. The deletion in progerin eliminates a ZMPSTE24 cleavage site, resulting in retention of farnesyl and methyl groups. Prelamin A that accumulates in cases of ZMPSTE24 deficiency also retains farnesyl and methyl groups due to lack of ZMPSTE24 cleavage (Figure created in Biorender.).

Shown in Fig. 2 is a schematic of the A-type lamin processing pathway as it is believed to occur within the nucleus [44,45]. In normal lamin A processing, a farnesyl (lipid) group is attached to the cysteine residue of the CaaX motif by a farnesyltransferase (FTase) [46]. The farnesyl group is a 15-carbon isoprenoid consisting of three isoprene units. The posttranslational addition of a farnesyl group, a type of prenylation, is important for binding of proteins to membrane surfaces [46]. Prenylation is followed by proteolytic cleavage of the three C-terminal amino acids of the CaaX motif (-aaX) by either of the integral membrane proteins Ras converting enzyme 1 (RCE1) or zinc metal-lopeptidase ste24 (ZMPSTE24), which both reside within the INM [44, 46–53]. After prenylation and the first cleavage event occurs, the cysteine residue of the CaaX motif is then methylated by an isoprenylcysteine methyltransferase (ICMT). These two modifications allow prelamin A to localize and anchor to the INM and enhance the metabolic stability of the protein [46,54,55]. A second cleavage reaction is then carried out specifically by ZMPSTE24 in which the last 15C-terminal amino acids, with the farnesyl and methyl groups attached, are removed to yield the mature lamin A protein [45,56]. As shown in Fig. 2, in the absence of ZMPSTE24 cleavage (which is the case in certain genetic disorders), the consequence is the accumulation of an incompletely processed form of lamin A which is permanently farnesylated and methylated since these modifications are irreversible [44]. These latter groups cause the incompletely processed lamin A to be trapped within the INM instead of localizing to the NL where mature lamin A should ultimately reside.

## 3. Genetic disorders arising from mutations that disrupt the NL

Mutations in lamin genes cause an extensive range of genetic disorders with tissue-specific phenotypes, an indication that lamins can interact with various binding partners that contribute to the tissue-specific disease phenotypes [22]. Mutations in genes that affect the expression or processing of a lamin protein, most commonly lamin A, are the cause of a group of genetic disorders known as laminopathies [38, 47]. Laminopathies can further be classified into primary laminopathies and secondary laminopathies [38]. Primary laminopathies are caused by mutations in the *LMNA* gene [38]. Secondary laminopathies are caused by mutations in genes coding for prelamin A processing proteins (such as ZMPSTE24), B-type lamins, and lamin-binding proteins [1]. Laminopathies cause a range of different diseases [38,57]. Hutchinson-Gilford Progeria Syndrome (HGPS), a type of primary laminopathy, is caused via a de novo C to T single-base substitution in the *LMNA* gene. The HGPS mutation activates a cryptic splice site in exon 11 which results in abnormal splicing of the *LMNA* gene to produce a transcript encoding a mutant form of lamin A known as progerin (Fig. 1). Progerin displays an internal in-frame deletion of 50 amino acids of the lamin A protein, and this deletion encompasses the ZMPSTE24 cleavage site [38]. As a result, progerin cannot be cleaved by ZMPSTE24 since the cleavage site is absent, and so progerin remains incompletely processed and retains the farnesyl and methyl groups (Fig. 2) [38]. HGPS patients appear healthy at birth, but clinically present within the first few years of life with features of premature aging [38,58,59]. These patients live to an average age of 14 due to heart attacks and congestive heart failure [58].

Reduction in ZMPSTE24 activity can also lead to disease, and it has been shown that the level of ZMPSTE24 activity may correlate with disease severity and phenotypes [57]. Restrictive dermopathy (RD) is an example of a severe secondary laminopathy disorder predominantly caused by compound heterozygous null mutations that create premature termination codons in the *ZMPSTE24* gene, causing a lack of functional ZMPSTE24 [60] and the attendant accumulation of farnesylated and methylated prelamin A (Fig. 2). This laminopathy is characterized by intrauterine growth restriction, tight rigid skin, small-sized mouth, thin hair, and bone mineralization defects [48]. This disease is neonatal lethal, and if patients survive until birth, they usually do not live past their first week of life due to pulmonary insufficiency [48]. Another secondary laminopathy disorder, known as atypical Hutchinson-Gilford Progeria Syndrome (AT-HGPS) or severe progeria, often involves compound heterozygous mutations within the *ZMPSTE24* gene consisting of a null allele and a hypomorphic allele preserving some ZMPSTE24 function [57]. AT-HGPS is also associated with premature aging [57]. Lastly, a rare autosomal recessive secondary laminopathy known as mandibuloacral dysplasia (MAD-B) is caused by compound heterozygous mutations in the *ZMPSTE24* gene that, like AT-HGPS, preserves some ZMPSTE24 function [57]. MAD-B is characterized by postnatal growth restriction, mandibular and clavicular acro osteolysis, delayed closure of the cranial suture, joint contractures, and lipodystrophy [38, 57,61–64]. Patients with RD, AT-HGPS, and MAD-B also display a misshapen nuclear envelope due to the lamin A/C imbalance that occurs since ZMPSTE24 activity is insufficient to appropriately process prelamin A to mature lamin A [65]. As described above, mutant alleles in RD have been shown to retain no activity, while alleles in AT-HGPS and MAD-B show some residual activity [57]. Therefore, the site of mutation within the *ZMPSTE24* gene could explain the diversified phenotypes that patients may present [66].

In addition to lamin A processing, ZMPSTE24 has other functions within the cell, including endoplasmic reticulum (ER) translocon pore clog clearing [67,68]. Translocons become clogged if proteins cross into the pore but cannot depart from it. When this happens, ZMPSTE24 cleaves the proteins into peptide fragments for clearance out of the pores [69]. *ZMPSTE24* mutations that impact lamin A processing are genetically distinct from mutations that effect the ability of ZMPSTE24 to clear proteins from clogged translocon pores [70]. ZMPSTE24 also interacts with the interferon-induced transmembrane protein family to facilitate these proteins in blocking entry of enveloped DNA and RNA viruses into the cell, including influenza A, Zika, and SARS-COV-2 [71–74]. ZMPSTE24 also has additional substrates, including non-prenylated proteins, but not much is known about these events [75]. These additional functions could also help explain the differing phenotypes and severity of disease correlated with ZMPSTE24 expression.

Not only can mutations impacting lamin proteins cause genetic disorders, but mutations in other NL associated proteins can cause genetic disorders as well. Alterations in proteins within the NL can lead to a wide variety of syndromes affecting many tissue types. Mutations in *EMD*, the gene encoding Emerin, a protein that is essential to chromatin organization and gene expression, causes X-linked Emery-Dreifuss muscular dystrophy [8,76]. This is a disorder that causes a nearly identical disease phenotype of contractures, deterioration of skeletal muscle and cardiomyopathy as caused by mutations in *LMNA* [77]. Dysregulation of MAN1, a protein that is involved in regulation of gene expression, can cause a variety of different disorders that impact the skin and bones [18, 76]. Loss of function mutations in the *LEMD3* gene encoding MAN1 can cause disorders including osteopoikilosis, nonsporadic melorheostosis, and Buschke-Ollendorff Syndrome [76]. These disorders mostly cause excessive bone growth and occasional skin abnormalities [78]. Mutations in *LBR*, the gene encoding the protein lamin B receptor, lead to disorders involving bone and many tissue types, including disorders such as Pelger-Huet anomaly and Greenberg skeletal dysplasia [76]. Pelger-Huet anomaly is characterized by hypolobulation and altered chromatin structure of neutrophil nuclei [79]. Greenberg skeletal dysplasia is caused by a truncation mutation that affects bone and causes a loss of activity in enzymes involved in cholesterol synthesis [80]. All isoforms of nesprins, proteins found within the ONM which interact with the NL, can also lead to a variety of different disorders that impact the central nervous system, striated muscle, and the inner ear [76]. These include muscular dystrophy/cardiomyopathy and high-frequency hearing loss [76]. All in all, proper functioning of the NL is vital to ensuring proper functioning of a variety of tissues in the human body, with mutations in genes encoding NL proteins producing disease phenotypes in numerous tissue types. Along with this, many disorders of the NL also lead to genomic instability.

## 4. Consequences of an altered NL on genome maintenance

Cells expressing farnesylated variants of lamin A have been shown to display an increase in the levels of DNA damage, including DNA double-strand breaks (DSBs), which may contribute to many of the phenotypic manifestations found in these cells [47,48,81–83]. A DSB occurs when the two strands of DNA each suffer a break within close proximity to one another [84]. DSBs can be generated by chemical or radiological insult, or they may form spontaneously from other DNA lesions or at stalled or collapsed replication forks. When a cell accrues DNA DSBs, it is essential to the genomic stability of the cell to repair that damage. A DNA DSB is one of the most severe types of DNA damage, as it can lead to mutations, gross genomic rearrangements, and/or cell death [84–86].

In addition to displaying an increased number of DSBs, cells derived from *ZMPSTE24* −/− mice, as well as fibroblasts derived from HGPS patients, display increased sensitivity to DNA damaging agents [87,88]. This sensitivity is associated with reported failure of recruitment of DNA repair proteins, including RAD51 and p53- binding protein 1 (53BP1), to sites of DSBs, indicating a disruption of DNA repair [87]. RAD51 is an important DNA repair protein that can bind to single-stranded DNA tails at DSBs and facilitate the homology search and strand invasion steps of the homologous recombination (HR) pathway for DSB repair (discussed further below) [87]. One way in which lamin A may impact DNA repair processes is through its known binding with RAD51, normally protecting it from ubiquitylation and subsequent proteosome-mediated degradation [89]. However, if this interaction does not occur or is weakened, such as may be the case in laminopathies, RAD51 may too readily be degraded and not be recruited to damage sites [87,89].

Normally, lamins create a structural scaffold for the nucleus to confer strength and shape to this organelle [2–5,22,23]. Accumulation of variants of farnesylated prelamin A in laminopathies results in profound alteration of nuclear structure, including blebbing and potential rupturing of the nuclear envelope [90–96]. Cells derived from RD and HGPS patients have been shown to display herniations in the NE with chromatin extrusion and DNA leakage into the cytoplasm [38,96], which can activate the cGAS-STING inflammation pathway [97]. NE interruptions can also accumulate during the normal aging process [59, 60,91,98–101]. Interestingly, in addition to playing an important role in DNA repair, RAD51 has been shown to be involved with protecting the cell from DNA leakage from the nucleus through RAD51’s ability to bind single-stranded DNA and double-stranded DNA [102]. Knockdown of RAD51 in HeLa cells and pharmacological inhibition of RAD51 in HT1080 cells using suberoylanilide hydroxamic acid has been shown to cause enhanced cytosolic leakage of DNA [103,104]. It is possible that reduced binding of lamin A with RAD51 in laminopathies and the subsequent destabilization of RAD51 may lead to a greater amount of DNA leakage from the nucleus. Leaks in the NE also disrupt cellular compartmentalization, potentially allowing cytosolic components to inappropriately enter the nucleus. It is entirely possible that the entry of certain cytosolic components into the nucleus may impart additional genomic damage.

As mentioned above, cells expressing altered forms of lamin A such as progerin also generally show a decrease in recruitment of 53BP1 to damage sites. 53BP1 is a DSB repair protein that blocks 5’ DNA end resection, which is vital in DNA repair pathway choice to promote canonical non-homologous end-joining (NHEJ) [105–107]. It has been shown by co-immunoprecipitation that lamin A interacts with 53BP1 and plays a role in recruitment of 53BP1 to sites of DNA damage [108]. During a DNA damage response, interactions between 53BP1 and lamin A are decreased as 53BP1 is delivered to the site of damage [109]. When progerin is present, 53BP1 can interact strongly with progerin, suggesting progerin is directly involved in interfering with the recruitment of 53BP1 to sites of DNA damage, potentially contributing to the increased accumulation of damage [108]. Although foci of RAD51 and 53BP1 are generally reduced in cells expressing progerin due to failure to recruit these proteins to sites of damage, an increase in the number of foci of DNA damage marker γH2AX has been found in cells with altered lamin A processing, diagnostic for the accumulation of DNA damage in these cells [88,100–112]. Additional phenotypes associated with HGPS include low growth potential of cells from HGPS patients, defective activation of DNA damage checkpoints, short telomeres, and defective nuclear pore organization [47,83,91,108,110–114].

## 5. Influence of an altered NL on DNA repair pathways: a closer look

**Fig. 3.**
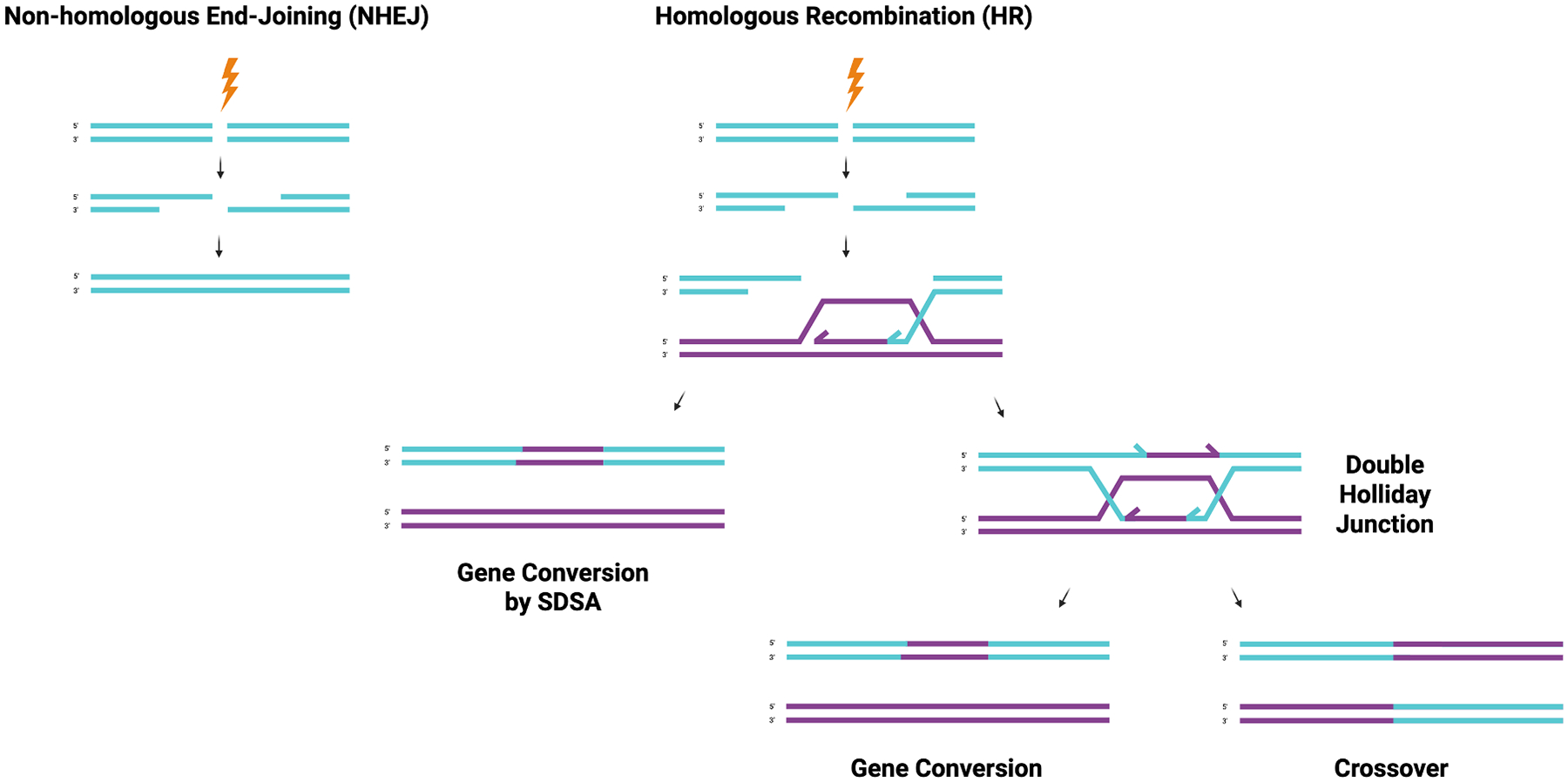
Schematic overview of DSB repair via NHEJ and HR. NHEJ involves the re-joining of DNA ends without the use of a repair template whereas HR uses a homologous sequence as a repair template. In HR, a 3’ DNA end (barbed DNA end in figure) from the DSB invades a homologous sequence and is extended off the invaded template. The HR event may proceed either with or without the formation of Holliday junction intermediates. In the absence of formation of Holliday junctions, as in the SDSA pathway, the extended invading strand is released from the homologous template and anneals to a DNA strand at other end of the break, leading to a gene conversion. If Holliday junctions are formed, either a gene conversion or a crossover may result depending on the manner by which the Holliday junctions are resolved. Details of Holliday junction resolution are not shown. (Figure created in Biorender.).

As alluded to above, one type of DNA damage that cells expressing farnesylated variants of lamin A have particular difficulty dealing with is the DSB [108,115,116]. Cells normally utilize two types of pathways to repair DSBs, HR and NHEJ (Fig. 3). HR plays a role in a variety of biological processes important to maintaining genomic stability. HR is an accurate form of DNA break repair that requires a template strand to repair the damaged DNA, while preserving and restoring information that may have otherwise been lost. HR occurs primarily during late S and the G2 phase of the cell cycle in dividing cells. DSB repair by HR can occur via two different modes, gene conversions (GC) and crossovers (CO) [117]. A GC occurs when two homologous sequences interact, and a relatively short segment of the damaged DNA gets converted to mirror the homologous sequence (Fig. 3). A CO occurs when the homologous sequences undergo a wholesale swap with each other at a point of exchange, as shown in Fig. 3. NHEJ is a less accurate form of DSB repair that joins DNA ends without a template strand [117,118]. There are several sub-pathways that can be classified under the umbrella term NHEJ, all sharing the feature of joining DNA ends without the use of a template. For simplicity, this family of DNA end-joining pathways will be referred to collectively as NHEJ. DSB repair via NHEJ, which can occur throughout the cell cycle and in nondividing cells, can produce insertions or deletions the site of the DSB, which can be detrimental to the cell [117,118].

Progerin expression in HGPS has been associated with DNA repair defects and studies have shown that recruitment of a host of HR repair proteins to DSBs, such as RAD50, RAD51, NBS1 and MRE11, are delayed [115,116]. As described above, the lack of recruitment of RAD51 may in part be due to destabilization of RAD51 by altered NL structure. Studies using extrachromosomal substrates in human cells have provided evidence that DSB repair associated with progerin expression shifts from HR to NHEJ [47,88,119–123], which might be expected if HR proteins are not being recruited to the DSB. There is some evidence that HR events may selectively occur at the nuclear envelope following migration of a chromosomal DSB to the nuclear periphery where it associates with certain components of the NL, most commonly with nuclear pore complexes in particular [124–127]. There is also evidence that, normally, specialized cytoplasmic microtubule structures might actually push the NL to the site of a DSB in order to prompt DSB repair [128]. If specialized sites on the NL do indeed serve as types of platforms or “factories” for DSB repair in general, or for certain DSB repair pathways specifically, such a scenario could further explain why an aberrant NL may shift the choice of DSB repair or corrupt repair in other ways.

We have developed a model system for studying DSB repair within mammalian chromosomes and we have used our system to investigate how defects in the NL impact DSB repair. This system involves the integration of a DNA repair substrate into a chromosome in cultured mammalian cells which allows us to study intrachromosomal DSB repair at the nucleotide level [129,130]. The repair substrate contains a recognition site for the endonuclease I-SceI. Following introduction of I-SceI into cells, we can use genetic selection to recover DSB repair events that occur via either accurate HR events between the broken sequence and a closely linked homologous sequence, or NHEJ events which display a deletion, or insertion, at the DSB site. Using our experimental system, we recently reported direct evidence that expression of progerin in otherwise normal human cells shifts repair of a genomic DSB from the HR pathway to the NHEJ pathway [129], consistent with earlier reports using extrachromosomal substrates [47, 88,119–123]. We also reported a decreased ability of a cell’s repair apparatus to accurately join closely spaced complementary single-stranded DNA tails when progerin is expressed [130]. We additionally found that progerin increases the size of deletions in NHEJ and reduces use of microhomology in end-joining [129,130]. Regarding HR events that did occur in cells expressing progerin, we noted a striking shift away from CO events and toward GC events [130]. This may reflect a progerin-induced alteration in the nature or strength of interaction between homologous sequences. It is possible, for example, that progerin-induced changes in chromatin structure and/or the set of proteins recruited to sites of repair allow DNA: DNA interactions sufficient for the “quick” strand invasion and release that may occur in GC events that proceed in the absence of Holliday junction formation as in the synthesis dependent strand annealing pathway (SDSA), but insufficient for a perhaps more prolonged interaction needed to complete formation of the Holliday junction intermediates required for CO (see Fig. 3). Like other groups, we have also observed that progerin expression causes an increase in the accumulation of spontaneous DSBs compared to cells not expressing progerin [131], which may explain our observation of an increased occurrence of spontaneous recombination in progerin-expressing cells in the absence of artificial DSB induction by I-SceI [129].

To summarize, progerin expression interferes with proper recruitment of DNA repair proteins to sites of damage, shifts DNA repair from HR to NHEJ, promotes HR repair via GC over CO, reduces precise joining of DNA ends, and reduces the use of microhomology in DNA end-joining. Overall, progerin expression has been shown to significantly impact the processes of DNA repair, which can lead to genomic instability.

## 6. The role of the NL in DNA replication

Along with alterations in DNA repair, cells from laminopathy patients have problems with DNA replication. Normal human cells engineered to express progerin, along with cells from HGPS patients, have been found to be sensitive to DNA damaging agents [88,108]. They are specifically sensitive to those that induce replication stress, which is any event that disrupts the advancement of a DNA replication fork [88,108]. It is hypothesized that lamin A normally recruits PCNA, DNA polymerase delta, and other proteins to replication forks by binding to the newly synthesized DNA strands where these proteins are needed. The mechanism of delivery of replication proteins is complex and not well understood [115,133–140]. However, progerin and farnesylated prelamin A expression have been shown to interfere with this recruitment, which can lead to replication fork stalling and collapse [115,133–142].

**Fig. 4.**
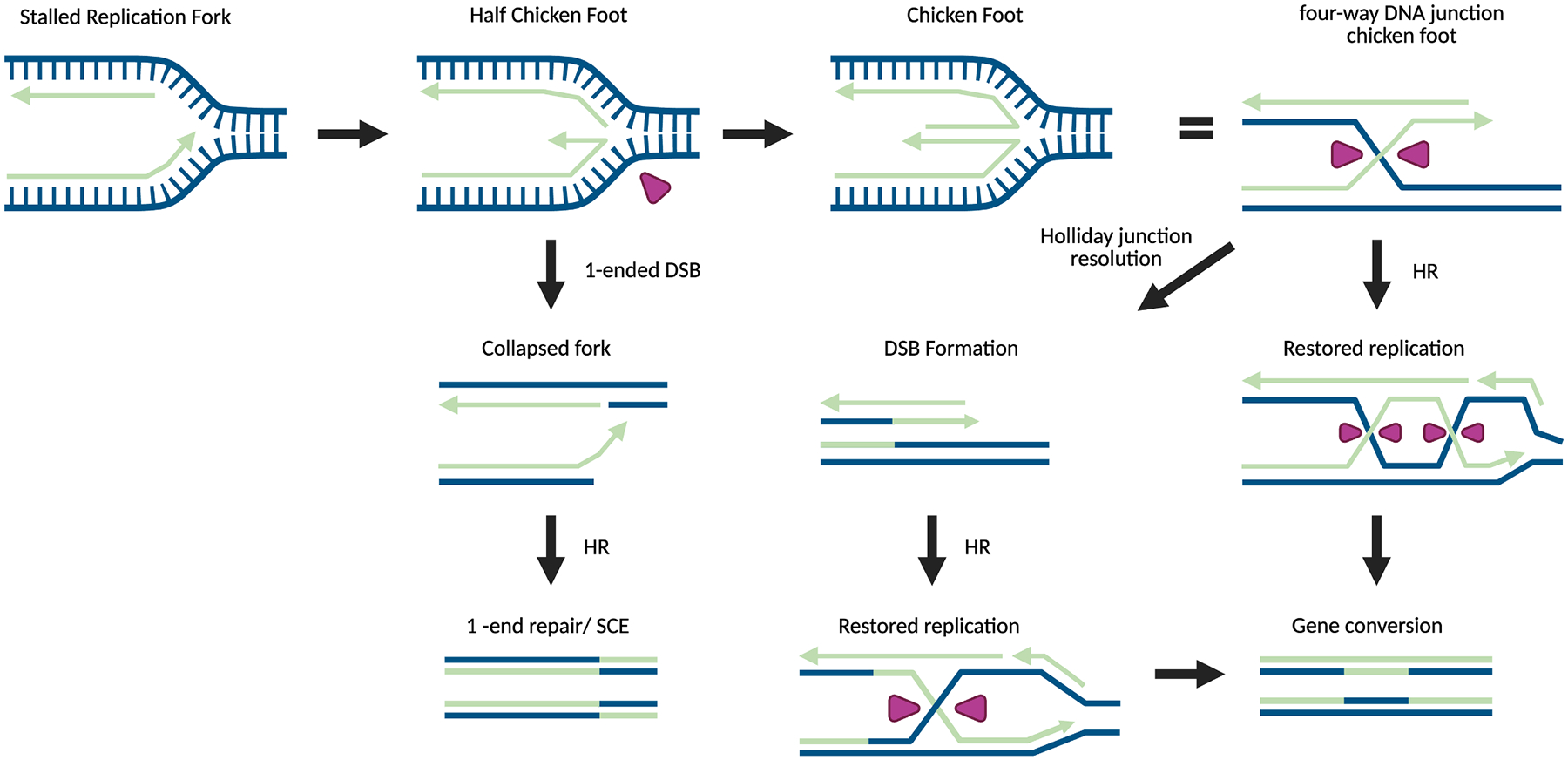
Mechanisms for recovery from stalled and collapsed replication forks. Magenta triangles indicate where breaks are made. “SCE” refers to sister chromatid exchange. The half chicken foot is the initial structure formed when a replication fork is reversed. Further replication fork reversal produces the chicken foot structure, serving as a substrate for HR proteins to act on, which can lead to replication fork rescue and continued replication. (This figure is adapted from Helleday [132] and was made in Biorender.].

Recovery from stalled or collapsed replication forks is normally accomplished by a variety of processes that are mechanistically equivalent to HR, as shown in Fig. 4. As discussed above, we and others have shown that progerin expression suppresses a cell’s ability to carry out HR [129,130], and so recovery from stalled and collapsed replication forks would be expected to present a particular challenge for cells expressing progerin. Further, a reduced availability of RAD51 in laminopathies presents additional challenges since RAD51 not only plays a role in the strand invasion/homology search step of HR but also promotes replication fork reversal [87]. An inability to effectively reverse stalled replication forks would likely enhance the susceptibility of stalled forks to collapse and nucleolytic degradation.

To summarize, defects in the NL may provoke the dual effects of interfering with both the progression of DNA replication forks as well as the efficient recovery from stalled replication forks. These problems would be expected to be a significant source of DNA damage, particularly DSBs, which can lead to genomic instability. To make matters worse, it has been shown that the nucleotide excision repair protein XPA mislocalizes to sites of DSBs at collapsed replication forks in cells expressing progerin, which could further hinder DNA repair since proper DSB repair proteins cannot localize to the site of a DSB if XPA is blocking such recruitment [135,136,138]. The mislocalization of XPA to stalled or collapsed replication forks is believed to be due to the affinity of XPA for junctions between single- and double-stranded DNA that are present at replication forks. Binding of XPA to replication forks is normally blocked by PCNA, which is not effectively recruited to replication forks in cells expressing progerin as discussed above [135,136,138].

In contrast (or in addition) to the generally accepted paradigm described above in which stalled replication forks serve as a main source of DNA damage in laminopathies, we have recently reported that progerin expression in immortalized cells can inflict genomic damage without impacting the ability of cells to proliferate or replicate [88]. Our studies therefore strongly suggest that there is a source of progerin-induced damage independent from stalled or collapsed replication forks, and thus also independent from mislocalization of XPA at breaks [88]. We did observe, however, that when cells expressing progerin are treated with hydroxyurea (HU), which reduces nucleotide pools and interferes with replication by inhibiting nucleotide reductase, the cells display particularly high levels of damage which is repaired very slowly [88]. Our results with HU thus confirm the idea that cells with an altered NL indeed have difficulties recovering from stalled replication forks should they occur.

An attractive hypothesis is that these latter observations regarding HU treatment may relate to limiting pools of dNTPs in cells expressing progerin. It has indeed been reported that dNTP pools are limiting in cells from HGPS patients [108]. This may be due in part to a reported alteration in replication timing that leads to an increased number of replication forks firing simultaneously that consequently depletes these pools and promotes replication fork stalling (discussed further below) [108]. HGPS cells also have impaired nucleotide metabolism, which can also contribute to these limited dNTP pools [143]. In our work involving immortalized cells expressing progerin, we obtained no evidence that replication forks were routinely stalling, but it is still possible that the cells were living on the edge with regard to availability of nucleotides due to the need for the cells to continually repair the elevated DNA damage that we monitored. If this were case, this would contribute to the particularly severe consequences of further reducing nucleotide pools with HU treatment. The persistence of damage inflicted by HU may of course also be associated with the difficulty that impaired HR presents for the recovery from stalled and collapsed replication forks that are predicted to occur upon HU treatment.

The notion that cells expressing progerin may be living on the edge of nucleotide availability may also help explain progerin’s impact on the very nature of DSB repair. As described earlier, cells expressing progerin display a shift in DSB repair pathway choice away from HR and toward NHEJ. Further, HR events are significantly shifted away from CO events and towards GC events. It is intriguing to speculate that these alterations in DSB repair pathways may be provoked by reduced nucleotide pools elicited by progerin expression, since the HR pathway requires more DNA synthesis than does NHEJ, and CO events most likely require more DNA synthesis than GC events

## 7. The impact of the NL on chromatin organization

The nucleus has limited space to complete its various functions; thus genomic DNA is folded into a more compact form. The nucleosome is the lowest level of compaction where DNA is wound around an octamer of histone proteins [144]. Histone proteins provide the structural basis for the first level of chromatin compaction and can further affect chromatin organization by attachment of chemical group modifications or by being replaced by histone variants [144]. Condensation of nucleosomes involves organization into a compact 30nm fiber, which can further be condensed with the help of nuclear proteins and divalent cations [145]. Any condensation beyond the 30nm fiber is known as higher order chromatin structure, eventually leading to the classical structure of chromosomes [144]. Modifications to histones influence binding of proteins to the nucleosome that can impact chromatin structure and gene expression [146]. Chromatin-binding proteins and histone modifications can lead to “open” and “closed” chromatin structure, which controls cellular processes like transcription and DNA repair [144]. Acetylation of histones is associated with open chromatin and transcription [147]. Methylation can activate or repress gene expression depending on the amino acid that is modified [147]. Phosphorylation is involved with the DNA damage response, DNA repair, chromatin compaction for mitosis and meiosis, and transcriptional activity [148, 149]. Ubiquitination can lead to protein degradation and altering of chromatin structure, which influences gene expression [147].

There are two main classes of chromatin, heterochromatin and euchromatin. Heterochromatin generally remains more tightly condensed, while euchromatin is more dynamic and in its relaxed state is associated with transcription [144,150]. Heterochromatin also has a different nuclear location than euchromatin and is primarily found at the nuclear periphery and around nucleoli [151]. This distribution of chromatin is viewed as a critical component of global genomic architecture [152,153], and the NE plays an important role in developing and preserving this architecture by serving as a stable structure to which chromatin can be attached [154,155]. Disturbance of local and global chromatin structure has been linked to several types of disease, including cancer [156].

One vital mechanism for managing genome folding in metazoan nuclei involves contacts between the genome and the NL [157–160]. These contacts occur within megabase-sized genomic regions termed lamina-associated domains (LADs), and the interactions between LADs and the NL play a role in global chromosome spatial organization. Genes contained within LADs are generally transcriptionally repressed and often show increased H3K9me3 levels [157]. Lamins have also been shown to bind DNA sequences termed matrix attachment regions which are sites of contact between DNA and the nuclear matrix, the three-dimensional filamentous protein network within the nucleoplasm that provides a framework for organizing chromatin structure and function [161,162]. The nuclear matrix facilitates nuclear processes such as DNA replication and gene expression.

Disruption of the NL can produce a host of significant impacts on the spatial organization and function of chromatin, with consequent impacts on overall nuclear structure. Cells expressing progerin have been found to have decreased chromatin condensation and stiff nuclei, which corresponds with the nuclear interior being less responsive to mechanical force to the nucleus [163]. HGPS patients are also known to show differences in higher-order chromatin organization, specifically changes in expression of chromatin-modifying proteins like HP1, involved in repressing gene expression [164,165]. Progerin expression in HeLa cells has been shown to create stiffer chromatin tethers that hold the chromatin to the lamina, which is believed to create the folds required for the formation of blebbed nuclei [166]. Primary HGPS skin fibroblasts have also been shown to display altered histone modifications that may result in transcriptional misregulation and trigger loss of spatial chromatin compartmentalization [167].

It has been shown that chromosome orientation in progerin-expressing cells has been shifted from centromeres facing the periphery to telomeres, with telomeres making more frequent contact with the lamina [108]. In the same study, progerin expression has also been shown to accelerate the rate of telomere shortening [108]. Fragile telomeres, which are telomeric regions where replication is impaired and can be visualized as multi-telomere signals using FISH that indicate a single chromosome end has more than one telomere signal, are found in progerin expressing cells [108]. These discoveries reveal telomere replication problems within progerin expressing cells and can provide an explanation for telomere length maintenance defects, which likely contribute to the accelerated aging phenotype observed in patients.

Lastly, a significant consequence of progerin expression is the loss of peripheral heterochromatin, the dense and transcriptionally repressed heterochromatin that is normally located adjacent to the NL [141,168, 169]. Heterochromatin usually replicates in late stages of the S phase. The loss of heterochromatin associated with progerin expression may lead to disruption of replication timing, resulting in these normally heterochromatic regions replicating earlier during S phase [141]. This may explain why cells expressing progerin have too many replication forks firing simultaneously, leading to the depletion of nucleotide pools. Interestingly, it has been reported that damage caused by progerin expression is inflicted specifically during S phase, and primarily during late S phase, consistent with the notion of cells running out of nucleotides [141,168,170]. It has also been reported that, significantly, supplementation of growth medium with nucleosides rescued the replicative signature seen in HGPS patient cells [108]. Whether corruption of DSB pathways elicited by progerin expression can also be partly or fully reversed by nucleotide supplementation awaits further exploration.

## 8. Final thoughts

Much work has been done studying genome maintenance in cells expressing progerin. However, it is not clear if ZMPSTE24 deficiency generated in an otherwise normal cell will produce the same effects as progerin expression, since farnesylated prelamin A which accumulates when a cell is depleted of ZMPSTE24 is not identical to progerin (progerin has a 50 amino acid deletion while prelamin A does not). Because ZMPSTE24 levels reportedly drop during normal aging [81,171], exploring the impact of ZMPSTE24 levels on cellular functions is of considerable interest. Learning how cellular pathways are altered by farnesylated prelamin A will help provide a foundation of knowledge for not only developing potential therapeutics for patients with *ZMPSTE24* mutations, but it will also provide insights into the biology of the normal aging process.

Although previous work using extrachromosomal DNA substrates has suggested that ZMPSTE24 deficiency results in impaired repair [47, 88,119–123], more work is needed to determine how ZMPSTE24 deficiency impacts the nature of repair of a DSB within a chromosomal context.

Knowledge gained by determining the impact that progerin expression or a drop in ZMPSTE24 expression has on genome stability and nuclear integrity can enhance our perspective on events associated with aging and age-related disease. Beyond basic biology, increased knowledge can lead to future investigations into ways of mitigating the impact that alteration to the NL has on genome integrity and subsequently on cellular functions. Our cell culture-based system for studying genomic DSB repair, or similar systems, can be directly employed in these future investigations. For example, such systems can be used to learn if treatment with farnesyl and/or methyl transferase inhibitors, or even nucleosides [108,170,172] will reverse corruption of DNA repair seen in cells expressing incompletely processed forms of lamin A. Also of considerable interest would be strategies for augmenting expression of repair/recombination/ replication proteins, such as RAD51, to attempt the rescue of altered DNA pathways and possibly combat DNA leakage from the nucleus into the cytosol [38,96,103,104]. The ultimate goal would be the development of treatments that show promise of counteracting the genome destabilizing effects of accumulation of altered forms of lamin A. Such therapeutic advances hold the promise of prolonging lifespan and health span for all.

## Figures and Tables

**Table 1:** Key Lamin Associated Proteins Within the NL and Their Proposed Functions.

Protein	Function
BAF	Influences higher-order chromatin structure and transcription repression [6,7]
Emerin	Binds A-type lamins and has roles in signaling, mechanotransduction, nuclear architecture, chromatin tethering and gene expression [8]
LAP1	Involved in maintaining proper nuclear envelope structure, chromatin positioning and cell migration [9,10]
LAP2	Binds lamin A exclusively in the nuclear interior to chaperone proper interaction with chromatin [11]
LAP2	Binds B-type lamins and provides docking sites for chromatin by interacting with histones [12]
LBR	Interacts with B-type lamins and provides a stable binding site for heterochromatin at the NE [13]
LEMD2	Maintains structural integrity and shape of the nucleus by interacting with BAF and linking to A-type lamins; involved in chromatin binding and distribution [14,15]
LINC Complex	Linker of nucleoskeleton and cytoskeleton (LINC), allowing for communication between the nucleus and cytoplasm [16,17]
MAN1	Involved in regulating gene expression [18]
Nucleoporins	Group of 30 different proteins that form the nuclear pore complex (NPC). Crucial for anchoring and proper function of NPCs in the NE [19,20]
Nurim	Contributes to nuclear envelope stability and inhibits apoptosis [21]

## Data Availability

No data was used for the research described in the article.
